# Flavonoids as Promising Neuroprotectants and Their Therapeutic Potential against Alzheimer's Disease

**DOI:** 10.1155/2022/6038996

**Published:** 2022-08-28

**Authors:** Tarun Minocha, Hareram Birla, Ahmad A. Obaid, Vipin Rai, P. Sushma, Chandan Shivamallu, Mahmoud Moustafa, Mohammed Al-Shehri, Ahmed Al-Emam, Maria A. Tikhonova, Sanjeev Kumar Yadav, Burkhard Poeggeler, Divakar Singh, Sandeep Kumar Singh

**Affiliations:** ^1^Department of Zoology, Institute of Science, Banaras Hindu University, Varanasi 221005, India; ^2^Department of Biochemistry, Institute of Science, Banaras Hindu University, Varanasi 221005, India; ^3^Department of Anesthesiology, Rutgers New Jersey Medical School, Newark 07103, USA; ^4^Laboratory Medicine Department, Faculty of Applied Medical Sciences, Umm Al-Qura University, Makkah, Saudi Arabia; ^5^Department of Biotechnology and Bioinformatics, School of Life Sciences, JSS Academy of Higher Education and Research, Mysuru, Karnataka 570015, India; ^6^Department of Biology, College of Science, King Khalid University, 9004, Abha, Saudi Arabia; ^7^Department of Botany and Microbiology, Faculty of Science, South Valley University, Qena, Egypt; ^8^Department of Pathology, College of Medicine, King Khalid University, Abha, Saudi Arabia; ^9^Department of Forensic Medicine and Clinical Toxicology, Faculty of Medicine, Mansoura University, Mansoura, Egypt; ^10^Laboratory of the Experimental Models of Neurodegenerative Processes, Scientific Research Institute of Neurosciences and Medicine, Timakov St., 4, Novosibirsk 630117, Russia; ^11^Johann-Friedrich-Blumenbach-Institute for Zoology & Anthropology, Faculty of Biology and Psychology, Georg-August-University of Göttingen-37073, Germany; ^12^School of Biochemical Engineering, IIT BHU, Varanasi 221005, India; ^13^Indian Scientific Education and Technology Foundation, Lucknow 221005, India

## Abstract

Alzheimer's disease (AD) is one of the serious and progressive neurodegenerative disorders in the elderly worldwide. Various genetic, environmental, and lifestyle factors are associated with its pathogenesis that affect neuronal cells to degenerate over the period of time. AD is characterized by cognitive dysfunctions, behavioural disability, and psychological impairments due to the accumulation of amyloid beta (A*β*) peptides and neurofibrillary tangles (NFT). Several research reports have shown that flavonoids are the polyphenolic compounds that significantly improve cognitive functions and inhibit or delay the amyloid beta aggregation or NFT formation in AD. Current research has uncovered that dietary use of flavonoid-rich food sources essentially increases intellectual abilities and postpones or hinders the senescence cycle and related neurodegenerative problems including AD. During AD pathogenesis, multiple signalling pathways are involved and to target a single pathway may relieve the symptoms but not provides the permanent cure. Flavonoids communicate with different signalling pathways and adjust their activities, accordingly prompting valuable neuroprotective impacts. Flavonoids likewise hamper the movement of obsessive indications of neurodegenerative disorders by hindering neuronal apoptosis incited by neurotoxic substances. In this short review, we briefly discussed about the classification of flavonoids and their neuroprotective properties that could be used as a potential source for the treatment of AD. In this review, we also highlight the structural features of flavonoids, their beneficial roles in human health, and significance in plants as well as their microbial production.

## 1. Introduction

AD is one of the most prevalent neurodegenerative disorders that affects millions of people all over the world [1]. The social and economic burden of AD is high and the number of cases is rising dramatically and may reach to 88 million by 2050 [2]. AD is attributed by several pathological manifestations including profound oxidative stress, synaptic connection loss, cumulative emergence of intracellular tau pathology, accumulation of extracellular amyloid beta (A*β*) plaques, and other aspects [3]. During the onset and progression of AD, various neurological dysfunctions take place that ultimately leads to loss of memory and cognitive deficits that cause interference in daily life of the individuals [4]. Plenty of significant resources have been exhausted to date in its treatment, but the overall results are still disappointing and challenging. AD occures due to disturbance of multiple pathway, therefore, the multi-target approche thruogh hole plant extract will be more promising among the possible therapies. Ongoing analyses have demonstrated that consumption of flavonoid-rich diet can actually improve mental health and cognitive ability in people [5–8]. Furthermore, several flavonoids have been accounted to limit the progression of AD, and this has been originating from their capacity to improve cognitive function in various preclinical models [8]. The flavonoid-rich foods like cocoa, citrus, green tea, and berry can be ascribed to the connections of flavonoids and their metabolites with various subcellular targets [9, 10]. Consequently, flavonoids thus exert their neuroprotective effects by maintaining the neuronal quality and prevent the development of AD, involved in cognitive improvement. Overall, this review summarizes the classification, physiochemical properties, hallmarks of AD, and pharmacological effects of flavonoids against the pathogenesis of AD. This review also includes the molecular pathways targeted by flavonoids to prevent the progression of AD and provide a deep understanding in research of flavonoids.

### 1.1. Flavonoid Overview

A broad and varied range of organic chemicals are produced by plants, the vast majority of which do not appear to be directly involved in growth and development. Historically, these compounds have been referred to as secondary metabolites specially flavonoids. During the last few years, a myriad of studies has ignited the attention of polyphenolic compounds in the treatment of AD because of their possible therapeutic applications [11]. Flavonoids belong to one of the diverse groups of plant polyphenols, and more than 10,000 flavonoids have been extracted to date from natural resources including wines, vegetables, restorative plants, and organic products [12]. Flavonoids have emerged as a promising leading molecule either alone or in association with other compounds for showing the effective plan and improvement as anti-AD drugs [13].

### 1.2. Flavonoids and Neuroprotection

Their incredible diversity, circulation, availability, and simple detachment have made them a class of potent candidates for AD therapy. Various in vitro as well as in vivo studies have been conducted defining the antioxidant and neuroprotective property of flavonoids. In addition multiple preclinical and clinical studies also reported the role of flavonoids in the prevention and treatment of cognitive dysfunction, learning, and memory deficits [8]. Acute or chronic administration of flavonoids crosses through the blood-brain barrier suggesting that these compounds can feasibly have a direct effect on the brain and hence could be used as a prophylactic agent [14]. Furthermore, natural and synthetic flavonoids have gained substantial attention not only because of their antioxidative, anti-inflammatory, and antiamyloidogenic properties [15] but also because of multiple range of pharmacological effects that ameliorate learning and memory in the AD patients [16]. Moreover, most of the flavonoids are accounted for limiting the movement of AD pathologies, suppress the psychological shortfalls, and viably improve psychological capacities in humans [17]. Besides that, a few specialists are engaged in the various periods of clinical preliminaries, but none of the antiamyloid medication is presently clinically accessible. Because of the harmfulness related to the utilization of accessible medications and their restricted restorative viability, the quest for new anti-AD drugs is as yet in progress [18].

## 2. Classification of Flavonoids

More than 10000 different flavonoids have been identified to date which have enormous therapeutic potential. Despite having great diversity, the classification of flavonoids has restricted into six groups based on their molecular structure [17]. These groups include flavonols (rutin and quercetin), flavanols (catechin, epicatechin, and epigallocatechin), isoflavones (genistein, daidzein, glycetin, and formanantine), anthocyanidins (cyanidin, malvidine, and delphinidine), flavanones (hesperetin and naringenin), and flavones (apigenin and luteolin) [19]. Among the above mentioned six classes, the flavones are the most common and abundant within the families and genera of the higher plants [20]. Besides that, flavonoids are separated from one another based on contrasts in the condition of oxidation/reduction and aglycon ring design. Moreover, based on the extent of hydroxylation of aglycon, positions of the hydroxyl groups, saturation of pyran ring, and differences in the derivatization of the hydroxyl groups are major differentiating features among the various classes of flavonoids [21].

## 3. Distribution of Flavonoids in Nature

Flavonoids are the essential components of the plant cells hence, ubiquitously distributed in green plants [22] (Table 1). Additionally, they are part of the human diet due to their abundance in vegetables, fruits, seeds, and beverages such as coffee, tea, and red wine, as well as in medicinal herbs [23]. Being ubiquitous in nature, these secondary metabolites have emerged as an indispensable tool in a variety of nutraceutical, pharmaceutical, medicinal, and cosmetic applications and impose benevolent effects on human health [24]. Different dietary source contains different type of flavonoids with a range of conc. Like *Capparis spinosa* contain kaempferol (59 to 247 mg/100 gm) and quercetin (45-519 mg/100 gm), fresh rosemary contains high amount of naringenin (24.86 mg/ml). Petroselinum crispum commonly know as parsley is native species to European region that contains high amount of apigenin (1774-13506?mg/100 gm) and isorhamnetin (331?mg/100 gm). Very few human studies reported regarding concertation of flavonoid intake during the regular diet or from dietary components, because its concertation varies from one region to another in the same product. Future research needed to focus on dietary concentration of flavonoids and their absorption in human body.

## 4. Hallmarks of Alzheimer's Disease

The neuropathological hallmarks of AD include positive lesions such as amyloid plaques, neurofibrillary tangles, and cerebral amyloid angiopathy and negative lesions such as neuronal and synaptic loss [29].

### 4.1. Amyloid Plaques

**Figure 1:**
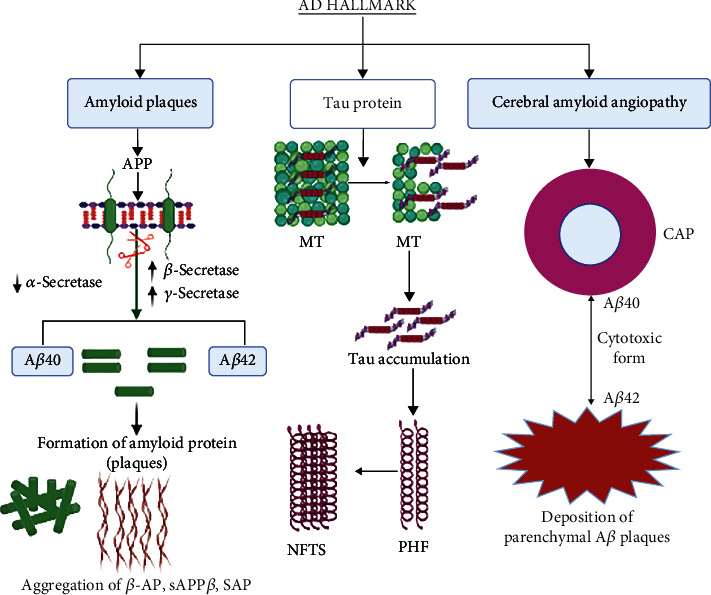
This figure depicts the overview of pathological hallmark of AD through amyloid plaque formation, NFT formation through Tau protein, and deposition of parenchymal A*β* plaques. APP: amyloid precursor protein; MT: microtubules; AD: Alzheimer's disease; NFTs: neurofibrillary tangles; PHF: paired helical filament.

Amyloid plaques also named senile plagues mainly composed of A*β* are one of the major hallmarks, and its accumulation leads to pathogenesis of AD [30]. Amyloid precursor protein (APP) is the precursor molecule in the generation of 37 to 49 amino acid residue peptide called A*β* with the help of *β*- and *γ*-secretase enzymes in neurons (Figure 1) [31]. The sequential cleavage leads to the deposition of two forms of A*β* with 42 or 40 amino acids (A*β*-40 and A*β*-42) and out of which A*β*-42 is more abundant and pathogenic as compared to A*β*-40 due to its insoluble nature [29]. In case of neurological disorders, the imbalance between A*β* formation and its clearance gets disrupted that ultimately leads to the formation of senile plaques [32]. An *in vivo* study reported that in the case of healthy people, the production rate of A*β* is lower than its clearance rate [33]; however, in the advancement of AD, the proportion of production and clearance gets disrupted in the brain.

### 4.2. Neurofibrillary Tangles

NFTs are considered to be another major pathological hallmark of AD. The ultraimages of AD brain revealed that NFTs are composed of fibrils of ~10 nm in diameter that form pairs with a helical conformation to give 65 nm paired helical filaments [34]. NFTs are found in both degenerated and dying neurons and composed of hyperphosphorylated tau protein (Figure 1). Tau is a microtubule-associated protein and its phosphorylated form is found in the somatodendritic region of neurons, and any abnormalities in its production lead to the production of NFTs and neuronal death [35]. The accumulation of NFTs is associated with neuronal dysfunction; that ultimately leads to dementia in AD patients.

### 4.3. Cerebral Amyloid Angiopathy (CAA)

CAA has been recognized as one of the morphologic hallmarks of AD, but its presence is also reported in the neurologically healthy brains of elderly patients (Figure 1) [36]. CAA results from deposition of *β*-amyloid in cerebral cortex region and the ultraimages of AD brain revealed that CAA is a major cause of pathologies in AD, including loss of smooth muscle cells, haemorrhage, capillary occlusion, and dementia in the elderly [37, 38]. We recently provided the first evidence that in a mouse model of CAA, oral administration of taxifolin, a natural bioactive flavonoid, prevented cognitive impairment through pleiotropic beneficial effects [39]. However, how CAA contributes to vascular dysfunction in AD is unclear, and effective treatments for CAA have not been established.

## 5. Biochemical Properties of Flavonoids

Flavonoids are of huge interest in numerous nutraceutical, pharmacological, medical, and cosmetic applications; they are a crucial ingredient. This is explained by their ability to control important cellular enzyme functions as well as their antioxidative [40], anti-inflammatory [41, 42], antimutagenic [43], and anticarcinogenic capabilities [44–46]. According to numerous researches, flavonoids possess biological properties such as antiallergenic, antiviral, anti-inflammatory, and vasodilation effects. These flavonoids can be found in a variety of foods, including cereals, tea, red wine, and fruits and vegetables [47]. The antioxidant activity of flavonoids, however, has drawn the greatest attention because of their capacity to both prevent the creation of free radicals and scavenge existing ones. Cellular damage brought on by oxidative stress has been linked to a number of diseases including diabetes, cancer, cardiovascular disease (CVD), neurological disorders, and ageing. Numerous biological molecules are harmed by oxidative stress, and the easy target is proteins and DNA which involved in every biological processes. Numerous researches have examined the ability of flavonoids to function as antioxidants in vitro, and significant structure-activity connections of the antioxidant activity have been identified [25] In the past years, researcher provided the molecular mechanisms of flavonoids to suppress the activities of specific P450 isozymes, in order to prevent carcinogens [48]. Recent research demonstrated that the flavonoids are also work as antiviral (e.g., quercetin, naringin, hesperetin, and catechin) and antibacterial (apigenin, along with quercetin) against the different kinds of infections [49, 50]. The effect of flavonoids, especially quercetin, is reported in the modulation of various inflammatory processes and immunological functions [51]. Tannins, stilbenes, curcuminoids, coumarins, lignans, quinones, and other flavonoids have chemopreventive properties as well as contribute to the induction of apoptosis by arresting the cell cycle, regulating carcinogen metabolism, and controlling ontogenesis expression, according to research by Ren et al. [48, 52]. They also mentioned that the flavonoids' potential mechanisms of action in the prevention of cancer include antioxidant activity and scavenging free radicals, modulation of carcinogen metabolism, control of oncogene and tumor-suppressor gene expression in cell proliferation and differentiation, induction of cell cycle arrest and apoptosis, and modulation of enzyme activities in detoxification. There is a lot of knowledge about flavonoids and how they protect the central nervous system, especially when it comes to neurodegenerative diseases brought on by the interaction of oxidative stress, inflammation, and transition metal accumulation. Like flavonols, flavonoids are linked to a decrease in the prevalence of dementia [53–55]. Citrus flavanones such hesperidin, hesperetin, and naringenin, according to Hwang et al. [56] and Wróbel-Biedrawa et al. and Silva dos Santos et al. [57, 58], may be able to cross the blood-brain barrier and be an effective treatment for neurodegenerative illnesses. It has also been claimed that of flavonoids have antiaging and antidiabetic properties [59, 60].

## 6. The Interaction of Flavonoids with Multiple Signalling Pathways in Alzheimer's Disease

**Figure 2:**
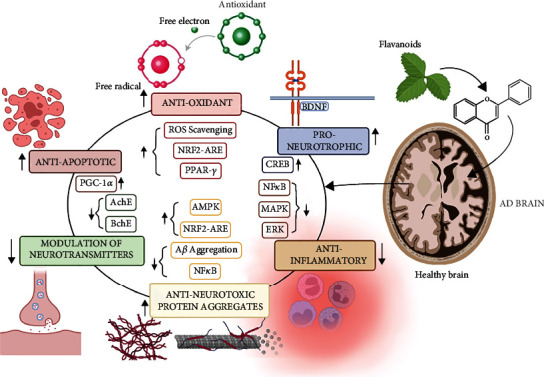
Role of flavonoids is regulation of different pathways. This figure shows flavonoids regulating various pathways including anti-inflammatory and antiapoptotic and play role as strong antioxidative agent to combat oxidative stress in Alzheimer's disease.

Various studies have revealed that flavonoids and their metabolites can exert useful actions on neurological processes through interactions with protein kinase and lipid kinase-signalling cascades [61]. These neuronal signalling pathways include nuclear factor kappa-light-chain-enhancer of activated B cells (NF-*κ*B) pathway (Figure 2) [62] as well as the protein kinase C (PKC), tyrosine kinase [63], phosphoinositide-3-kinase (PI3K)/Akt [64], and mitogen-activated kinase (MAPK) signalling pathways [16]. MAPKs are found to regulate a variety of cellular processes by converting extracellular signals into intracellular responses [65]. Flavonoids and their metabolites, on the other hand, can selectively interact with MAPK signalling pathways by interacting with MAPK kinases such as MAP kinase 1 (MEK1), MEK2, and membrane receptors (Figure 2) [66]. These kinases appear to promote the effect of flavonoids on the extracellular signal-regulated kinase (ERK) pathway [67]. Flavonoids have a structural similarity to a number of pharmacological ERK signalling pathway modulators [68]. Activation of the cAMP response element-binding protein (CREB) has also been observed as a result of ERK activation, which can result in upregulation of neuroprotective pathways as well as changes in memory and synaptic plasticity [69]. The nuclear factors erythroid 2-related factor 2 (Nrf2) and hypoxia-inducible factor-1, which act as modulators of PPAR-*γ* and activate the PGC-1*α* pathway, can be stabilised by flavonoids [70]. Flavonoids modify these molecular pathways, slowing the development of Alzheimer's disease by reducing oxidative stress, improving mitochondrial dysfunction, lowering insulin resistance, and improving memory impairment [71]. In addition, flavonoids also regulate PI3K/Akt signalling cascade through direct interactions with their adenosine triphosphate (ATP) binding site. In a study, it has been reported that the flavanone hesperetin has been found to cause activation of the Akt/protein kinase B (PKB) signal transduction cascade to provide prosurvival attributes in cortical neurons [72], while the flavonol quercetin was found to modulate the prosurvival Akt/PKB and ERK1/2 signalling cascade by hampering the activity of PI3K (Figure 2) [73]. Furthermore, it appears that cellular responses differ depending on the degree of interaction with downstream kinases or receptors, implying the possibility of structure-dependent signalling pathways.

Autophagy is a key process that is involved in synaptic plasticity and clearance of aggregate prone proteins in neuronal cells and aberrant autophagy plays a crucial role in neurodegenerative disorders [74]. Currently, flavonoids are being executed as a potential therapeutic tool for autophagy signalling involved in neurodegenerative disorders [75]. The group of few researchers reported that high dose of genistein (150 mg/kg/day) triggered the autophagy induction and promoted the degradation of A*β* and hyperphosphorylated tau in a streptozotocin-induced rat model of the sporadic AD [76]. In another study, it was found that resveratrol, a flavonoid, activated the first step of autophagy through AMPK/mTORC1 pathway to mitigate cognitive impairment in AD mice. Quercetin, the most potent flavonoid, not only cleared the A*β* aggregates but also delayed paralysis via activation of macroautophagy and limit brain damage *in vitro* and *in vivo* [77]. An *in vitro* study in SH-SY5Y neuronal cells revealed that flavonoid, wogonin, not only reduced the secretion of A*β* in the primary cortical astrocytes but also promoted its clearance through mTOR/autophagy signalling pathway [78]. Silibinin, another flavonoid, has also alleviated neuronal damage by inhibiting autophagy in the hippocampus region of the brain [79]. In addition, the combination of quercetin with silibinin and wogonin have been successfully used in preclinical and clinical studies to clear out the amyloid substance via induction of autophagy by ULK1/mTOR pathway [80]. Some studies reported on dietary intake of flavonoid and their benefits in AD patients (Figure 2) (Table 2).

## 7. Role of Flavonoids in Neuroprotection

Recently, flavonoid derivatives are being used as a neuroprotective agent in several neurodegenerative disorders including AD. The various aspects of flavonoids as a neuroprotectants are as follows.

### 7.1. Flavonoids as Neuroprotective Agents

**Figure 3:**
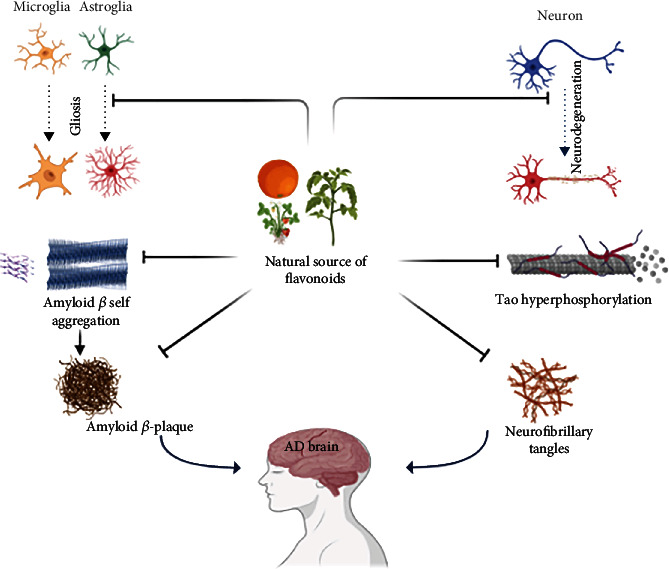
The neuroprotective role of flavonoids in inhibiting different signalling pathways responsible for Alzheimer's disease.

Flavonoids exert a multiplicity of neuroprotective effects against neurodegenerative diseases to suppress neuroinflammation and to improve cognitive function (Figure 3) [87–89]. Recently, one study from Gu et al. showed antiamyloid property of the flavonoid prepared from the caper leaf and fruit [90]. Another study from the Szwajgier conferred the neuroprotective effect of a flavonoid (Icariin) derived from the Chinese herb *Epimedium brevicornum*. Szwajgier showed the ability of icariin to decrease both amyloids-*β* (A*β*) and APP levels and increase neurogenesis in the brain of Tg2576 mice significantly [91]. Another flavonoid baicalein (5,6,7-trihydroxy-2-phenyl-chromen-4-one) extracted from *Scutellaria baicalensis*, has strong neuroprotective potential and significantly rescues synaptic plasticity and memory deficits in AD mouse model [92]. In addition, baicalein is able to prevent the damage in the hippocampal long-term potentiation (LTP) induced by A*β* and improve cognitive deficit associated with AD. People have also studied the anticholinesterase activity of few flavonoids [93], and as we know, there is increased activity of cholinesterase in case of AD so flavonoid having anticholinesterase activity will be useful to protect the neuronal damage in the hippocampus area of the AD patient.

### 7.2. Flavonoids as Cholinesterase Inhibitors

Acetylcholine (ACh) is the most versatile neurotransmitter that plays its role in transmission of impulse across different neurotransmitters. Cholinesterase enzymes including acetylcholinesterase (AChE) and butyrylcholinesterase (BChE) are completely engaged in the degradation of ACh responsible for the neurotransmission among synapses [94, 95]. A variety of studies revealed the low levels of ACh in AD brains and cholinesterase inhibitors not only increase ACh levels but also conduct the transmission of impulse at synaptic junctions [96]. Uriarte-Pueyo and Calvo summarized and represented 128 flavonoids exhibiting AChE-inhibiting activity making flavonoids anti-Alzheimer agents [97]. A myriad of flavonoid has been employed as inhibitors of AChE, but among them, the quercetin was found to be potent inhibitor of AChE [98]. The information in regard to the presently accessible medications demonstrates that utilizing this methodology is the most successful remedy in AD indicative treatment, along these lines smoothing out the inevitable clinical endorsement of four medications [99]. Attributable to the undesirable impacts and restricted adequacy of the presently accessible medications, there is a critical need to grow more protected and compelling medications [100–102]. In view of their intensity as AChE inhibitors, they were viewed as promising remedial specialists in the improvement of new enemy of Alzheimer drugs [103, 104].

### 7.3. Flavonoids as Cognition Enhancers

Flavonoids have been shown to reverse cognitive impairments and inhibit the progression of Alzheimer's disease, implying that they may have therapeutic potential [105]. Flavonoid-rich diets including grape juice, cocoa, and blueberry are used as memory enhancers [82, 106, 107]. In a randomized controlled clinical study, daily ingestion of anthocyanin-rich cherry juice improved fluency, short-term memory, and long-term memory in aged people (70+) with dementia [108]. Administration of quercetin along with daily usage of flavonoid-rich fruits increases cognition, memory retention, and recovery of short- and long-term memory loss [109].

Various studies have looked at flavonoids' antiamyloidogenic effects as a potential natural treatment for Alzheimer's disease. Moreover, flavonoids' effectiveness in learning and memory has been documented in a number of studies [110]. However, there are fewer studies on cognition and memory in animal models fed with fruits with high flavonoid-rich diet sources [111], e.g., anthocyanin-rich mulberry extracts corrected the cognitive impairment in mice with accelerated senescence and AD-like neurodegeneration [112]. A metanalysis revealed that dietary flavonoids improved the cognition function and showed its positive effect against AD pathogenesis [7]. Moreover, several recent studies reported on the beneficial effects of anthocyanin-enriched extracts or anthocyanin-enriched wheat grain diet on cognitive function in mouse AD models [113–115]. In a transgenic AD mouse model, a citrus flavonoid called nobiletin was found to reduce the strain of A*β* and plaques in the hippocampus, thus enhancing memory deficits caused by A*β* [116]. In both human Swedish mutant APP transgene-bearing neuron-like cells and primary neurons, the citrus flavonoid luteolin was found to minimise APP processing by amyloidogenic *γ*-secretase activity and decrease the generation of A*β*. Furthermore, the accumulation of A*β* in the brains of AD mouse models was prevented by administering curcumin or polyphenol-rich grape seed extracts for nine months [117]. However, further research is required to determine which flavonoid structures have potent beneficial properties as well as their underlying mechanisms of action. Three structural features of natural products have been suggested in a recent study to clarify their inhibitory function against A*β*-42 aggregation [118]. The first feature is carboxy acid derivatives of anthraquinonoids or triterpenoids that can form a salt bridge with basic amino acid residues like Lys28 and Lys16 in A*β*-42 trimers or dimers [119]. The second property is that noncatechol-type flavonoids have molecular planarity due to *αβ*-unsaturated carbonyl groups that can interact with the intermolecular *β*-sheet region in A*β*-42 aggregates, especially aromatic rings like Phe20 and Phe19 (Figure 3) [120]. Catechol-type flavonoids can form Michael adducts with the side chains of Lys28 and Lys16 in monomeric A*β*-42 through flavonoid autoxidation, which is the third characteristic [121].

### 7.4. Flavonoids as Free Radical Scavenger

Flavonoids possess several biochemical properties, but the most important biological role of flavonoids has been reported to have antioxidant and hydrogen-donating capacity [122]. The functional groups associated with the structure are prominently responsible for the antioxidant property of flavonoids. Oxidative stress is generated because of the imbalance between reactive oxygen species (ROS) and antioxidants and one of the important events in any neurodegenerative disorders. Apart from neurodegenerative disorders, oxidative stress has been linked to various other diseases including ischemic injury, cancer, atherosclerosis and inflammation. Oxidative stress and metal toxicity played a crucial role in the pathogenesis of AD [55]. Flavonoids play a crucial role in preventing the adverse effect of ROS by protecting the neurons against oxidative stress and hence suppress neuronal damage and improve cognitive function [20] [123]. Besides flavonoids, its derivatives have been reported to have potential antioxidant and neuroprotective activity *in vitro* and *in vivo* [89, 124–127]. Various studies have suggested that flavonoids exert its action especially in neuroprotection against AD by affecting gene expression and interacting with mitochondria and the intracellular signalling modulation of cascades that control neuronal differentiation, survival, and death [73]. Moreover, being a potent scavenger molecule, flavones exert an important indirect antioxidant activity contributing to the homeostasis of metal chelation, enzymatic activity modulation, and stabilization of membranes through antilipid peroxidation [122, 128]. Apart from the antioxidant activity of the flavonoids, these polyphenolic compounds also play very important role in human health and exhibit several other significant properties including antimicrobial, antiviral, and anticancer [129]. Antioxidant mechanism of the flavonoids reduces the free radical load and improves the health status through activation of antioxidant enzyme system that scavenges the reactive oxygen species (ROS) [130].

## 8. Demerits of Flavonoids

The wide accessibility of flavonoids and their new expansion in utilization by people has brought up significant issues with respect to the expected harmfulness of these dietary parts [131]. Albeit most of characteristic items are all around endured; nonetheless, flavonoids and related phytochemicals have been appeared to incite neurobehavioural and endocrine-disturbing impacts [132]. It has been stated that the quercetin possesses very low toxicity for humans and doses of approximately 1 mg/adult/per day have been recommended [133]. It has been reported that long-term consumption of quercetin over several years resulted in the formation of tumors in mice [134]. Afterwards, several studies were conducted but no study had reported the carcinogenicity and other ill effects of flavonoids [135]. The collaborations of flavonoids with CYP3A4, the prevalent human hepatic and intestinal CYP liable for processing half of restorative specialists, are exceptionally compelling. The concurrent organization of flavonoids and clinically utilized medications may cause drug-flavonoid interactions by balancing the pharmacokinetics of specific medications [133]. Dosage also seems to be an essential issue. For example, high doses of resveratrol caused various side effects including nausea, diarrhea, and abdominal discomfort [136]. Moreover, some flavonoids such as resveratrol or epigallocatechin-3-gallate are regarded as pan-assay-interference (PAINS) compounds [137]. Their effect on an organism might be nonspecific, including cell membrane perturbations, rather than specific protein binding [138], especially while supraphysiological doses are applied. Very few studies reported on side effect of flavonoids. Early studies reported that flavonoids (quercetin) act as mutagenic and cause toxicity for gene expression [139, 140]. Hodnick et al. observed that myricetin and quercetin caused mitochondrial enzyme inhibition, damage endogenous antioxidant, and accelerate oxidative stress [141]. Some flavonoids also inhibit topoisomerase II, although this help to recover the cancer patients, but beside this, it also increases the risk of leukemia. Further studies needed to further validate this result. Flavonoids also found to interfere with thyroid hormone production in infant with autoimmune disease. But no studies reported regarding flavonoids and thyroid production in healthy population. Tons of data present regarding role of flavonoids in treatment of various disease but still very few clinical studies carried out. Beside this, the rate of translation of preclinical to clinical studies is very less.

## 9. Future Prospective

During the last decade, flavonoids received much attention and a variety of beneficial effects have been elucidated against a myriad of neurodegenerative disorders. Further research is needed to discover new flavonoids from nature's bounty so that this may replace using synthetic drugs which are harmful to the body. In this context, there is a need of studies and development programmers related to in vivo studies so one can provide a hopeful and safe picture for the destiny. Presently, the consumption of fruit, greens, and drinks containing flavonoids is recommended, although it is too early to make hints on every day flavonoid intakes. Further studies are needed especially well-designed clinical trials to endorse the clinical effectiveness of flavonoids in neurodegeneration so that it might be used as a better prophylactic agent to improve the human health including AD patients. Another issue that needs to be addressed in the flavonoid research is to identify the amount of flavonoids absorbed, either they work in synergistic way or individual in improvement of human health.

## 10. Conclusion

This review will provide a brief insight about the pathology of AD and potential of natural flavonoids and their derivatives as active agents against neurological diseases. This should encourage research community in a new direction to exploit such information for the rational design of flavonoid based pharmaceutical drugs for AD. Flavonoid is very vast and present in almost natural sources with range of concentration. Various preclinical/clinical studies reported that flavonoids are effective against various diseases including AD. Further research needed especially clinical studies to standardise the dose, side effect, and bioavailability of flavonoids in AD patients.

## Figures and Tables

**Table 1:** Classification of flavonoids and their dietary sources.

S. no.	Class	Flavonoids	Dietary sources	References
1.	Flavanones	Naringin	Cabbage, banana, kiwi, garlic, olives, onion, sprout vegetables, lemon	[25]
		Naringenin		
		Hesperetin		
		Eriodictyol		
2.	Flavanols	Epigallocatechin gallate	Green tea, red wine, grapes, cocoa beans, apricots, berries, apple	[25]
		Epigallocatechin		
		Epicatechin		
		Catechin		
3.	Flavones	Luteolin	Kiwi, green tea, oregano, spinach, lettuce, broccoli, watermelon, peas, chamomile flower, orange, grapes, pumpkin, chickpea, brown rice, rosemary	[26]
		Diosmin		
		Apigenin		
		Wogonin		
4.	Flavonols	Quercetin	Green pea, grape seeds, apple, citrus fruits, soyabean, onions, cucumber strawberries, tomatoes	[26]
		Morin		
		Galangin		
		Kaempferol		
5.	Anthocyanins	Malvidin	Berries (red, purple and blue), red grapes, fruits (pomegranate, red apple, apricot), vegetables (black beans, red cabbage, purple carrot, eggplant, coloured potatoes, red onions, radish), coloured cereals	[27, 28]
		Cyanidin		
		Hirsutidin		
		Pelargonidin		
6.	Isoflavones	Genistein	Soybeans, soy foods, legumes, parsley, tofu, fava beans, red clover	[27]
		Glycitein		
		Equol		
		Daidzein		

**Table 2:** Role of flavonoids in Alzheimer's disease.

S. no.	Flavonoids	Study model	Reported dose	Route of administration	Natural source	Outcome of the studies	Reference
1.	Epigallocatechin-3-gallate	APPswe/PS1dE9 mice	40 mg/kg	Oral	Green tea	Decreases A*β* plaques, neuroinflammation, increases memory and learning	[81]
2.	Cocoa flavonols	Human	550 mg, 994 mg	Oral	Dark chocolate	Improve cognitive function	[82]
3.	Apigenin, luteolin, kaempferol, quercetin	Human	2 gm to 20 gm	Oral	Parsley	Bioavailability, antioxidative, and biomarker	[83] [84]
4.	Hesperidin, neohesperidin, naringenin, quercetin, rutin, etc.	Human	3-5 citrus per week	Oral	Citrus	Lower the risk of dementia	[85]
5	Flavanone	Human	Daily for 8 weeks	Oral	Orange	Cognitive benefits	[86]
6.	Combination of quercetin and dasatinib	Clinical trial in AD patients	—	Oral	Apple, honey, onion, citrus fruits	—	NCT04063124, NCT04785300, NCT04685590
7.	Epigallocatechin gallate	Clinical trial on AD patients	—	Oral	Tea, fruits, nuts		NCT03978052

## References

[1] Hickman R. A., Faustin A., Wisniewski T. (2016). Alzheimer disease and its growing epidemic: risk factors, biomarkers, and the urgent need for therapeutics. *Neurologic Clinics*.

[2] Kaur R., Sood A., Lang D. K. (2022). Potential of flavonoids as anti-Alzheimer’s agents: bench to bedside. *Environmental Science and Pollution Research*.

[3] Kabir M. T., Uddin M. S., Begum M. M. (2019). Cholinesterase inhibitors for Alzheimer's disease: multitargeting strategy based on anti-Alzheimer's drugs repositioning. *Current Pharmaceutical Design*.

[4] Brown R. C., Lockwood A. H., Sonawane B. R. (2005). Neurodegenerative diseases: an overview of environmental risk factors. *Environmental Health Perspectives*.

[5] Macready A. L., Kennedy O. B., Ellis J. A., Williams C. M., Spencer J. P. E., Butler L. T. (2009). Flavonoids and cognitive function: a review of human randomized controlled trial studies and recommendations for future studies. *Genes & Nutrition*.

[6] Socci V., Tempesta D., Desideri G., De Gennaro L., Ferrara M. (2017). Enhancing human cognition with cocoa flavonoids. *Frontiers in Nutrition*.

[7] Cheng N., Bell L., Lamport D. J., Williams C. M. (2022). Dietary flavonoids and human cognition: a meta-analysis. *Molecular Nutrition & Food Research*.

[8] Ramezani M., Meymand A. Z., Khodagholi F. (2022). A role for flavonoids in the prevention and/or treatment of cognitive dysfunction, learning, and memory deficits: a review of preclinical and clinical studies. *Nutritional Neuroscience*.

[9] Khan J., Deb P. K., Priya S. (2021). Dietary flavonoids: cardioprotective potential with antioxidant effects and their pharmacokinetic, toxicological and therapeutic concerns. *Molecules*.

[10] Flanagan E., Müller M., Hornberger M., Vauzour D. (2018). Impact of flavonoids on cellular and molecular mechanisms underlying age-related cognitive decline and neurodegeneration. *Current Nutrition Reports*.

[11] Kelsey N. A., Wilkins H. M., Linseman D. A. (2010). Nutraceutical antioxidants as novel neuroprotective agents. *Molecules*.

[12] Santos E. L., Maia B. H. L. N. S., Ferriani A. P., Teixeira S. D. (2017). Flavonoids: classification, biosynthesis and chemical ecology. *Flavonoids-From Biosynthesis to Human Health*.

[13] Bukhari S. N. A. (2022). Dietary polyphenols as therapeutic intervention for Alzheimer’s disease: a mechanistic insight. *Antioxidants*.

[14] Elbaz A., Carcaillon L., Kab S., Moisan F. (2016). Epidemiology of Parkinson's disease. *Revue Neurologique*.

[15] de Andrade Teles R. B., Diniz T. C., Costa Pinto T. C. (2018). Flavonoids as therapeutic agents in Alzheimer’s and Parkinson’s diseases: a systematic review of preclinical evidences. *Oxidative medicine and cellular longevity*.

[16] Uddin M., Kabir M. T., Niaz K. (2020). Molecular insight into the therapeutic promise of flavonoids against Alzheimer’s disease. *Molecules*.

[17] Harborne J. B. (2013). *The flavonoids: advances in research since 1980*.

[18] Rice-Evans C. (2001). Flavonoid antioxidants. *Current Medicinal Chemistry*.

[19] Falcone Ferreyra M. L., Rius S., Casati P. (2012). Flavonoids: biosynthesis, biological functions, and biotechnological applications. *Frontiers in Plant Science*.

[20] Heim K. E., Tagliaferro A. R., Bobilya D. J. (2002). Flavonoid antioxidants: chemistry, metabolism and structure-activity relationships. *The Journal of Nutritional Biochemistry*.

[21] Ayaz M., Sadiq A., Junaid M. (2019). Flavonoids as prospective neuroprotectants and their therapeutic propensity in aging associated neurological disorders. *Frontiers in Aging Neuroscience*.

[22] Havsteen B. H. (2002). The biochemistry and medical significance of the flavonoids. *Pharmacology & Therapeutics*.

[23] Mohebali N., Shahzadeh Fazeli S. A., Ghafoori H. (2018). Effect of flavonoids rich extract of Capparis spinosa on inflammatory involved genes in amyloid-beta peptide injected rat model of Alzheimer's disease. *Nutritional Neuroscience*.

[24] Abou Baker D. H. (2022). An ethnopharmacological review on the therapeutical properties of flavonoids and their mechanisms of actions: a comprehensive review based on up to date knowledge. *Toxicology Reports*.

[25] Panche A. N., Diwan A. D., Chandra S. R. (2016). Flavonoids: an overview. *Journal of Nutritional Science*.

[26] Hostetler G. L., Ralston R. A., Schwartz S. J. (2017). Flavones: food sources, bioavailability, metabolism, and bioactivity. *Advances in Nutrition*.

[27] Beecher G. R. (2003). Overview of dietary flavonoids: nomenclature, occurrence and intake. *The Journal of Nutrition*.

[28] Hornedo-Ortega R., Rasines-Perea Z., Cerezo A. B., Teissedre P. L., Jourdes M. (2022). *Anthocyanins: dietary sources, bioavailability, human metabolic pathways, and potential anti-neuroinflammatory activity*.

[29] Serrano-Pozo A., Frosch M. P., Masliah E., Hyman B. T. (2011). Neuropathological alterations in Alzheimer disease. *Cold Spring Harbor Perspectives in Medicine*.

[30] Sajjad R., Arif R., Shah A. A., Manzoor I., Mustafa G. (2018). Pathogenesis of Alzheimer's disease: role of amyloid-beta and hyperphosphorylated tau protein. *Indian Journal of Pharmaceutical Sciences*.

[31] Nunan J., Small D. H. (2000). Regulation of APP cleavage by *α*-, *β*-and *γ*-secretases. *FEBS Letters*.

[32] Harkany T., Ábrahám I., Timmerman W. (2000). *β*-Amyloid neurotoxicity is mediated by a glutamate-triggered excitotoxic cascade in rat nucleus basalis. *European Journal of Neuroscience*.

[33] Bateman R. J., Munsell L. Y., Morris J. C., Swarm R., Yarasheski K. E., Holtzman D. M. (2006). Human amyloid-*β* synthesis and clearance rates as measured in cerebrospinal fluid in vivo. *Nature Medicine*.

[34] Wiśniewski H. M., Narang H. K., Terry R. D. (1976). Neurofibrillary tangles of paired helical filaments. *Journal of the Neurological Sciences*.

[35] Kimura T., Yamashita S., Fukuda T. (2007). Hyperphosphorylated tau in parahippocampal cortex impairs place learning in aged mice expressing wild-type human tau. *The EMBO Journal*.

[36] Smith E. E., Greenberg S. M. (2009). *β*-Amyloid, blood vessels, and brain function. *Stroke*.

[37] Viswanathan A., Greenberg S. M. (2011). Cerebral amyloid angiopathy in the elderly. *Annals of Neurology*.

[38] Yamada M. (2015). Cerebral amyloid angiopathy: emerging concepts. *Journal of Stroke*.

[39] Inoue T., Saito S., Tanaka M. (2019). Pleiotropic neuroprotective effects of taxifolin in cerebral amyloid angiopathy. *Proceedings of the National Academy of Sciences*.

[40] Nurcholis W., Putri D. N. S. B., Husnawati H., Aisyah S. I., Priosoeryanto B. P. (2021). Total flavonoid content and antioxidant activity of ethanol and ethyl acetate extracts from accessions of Amomum compactum fruits. *Annals of Agricultural Sciences*.

[41] Medrano-Jiménez E., Meza-Sosa K. F., Urbán-Aragón J. A., Secundino I., Pedraza-Alva G., Pérez-Martínez L. (2022). Microglial Activation in Alzheimer's Disease: The Role of Flavonoids and MicroRNAs. *Journal of Leukocyte Biology*.

[42] Daily J. W., Kang S., Park S. J. B. (2021). Protection against Alzheimer's disease by luteolin: role of brain glucose regulation, anti-inflammatory activity, and the gut microbiota-liver-brain axis. *Biofactors*.

[43] Nemoto H., Otake M., Matsumoto T. (2022). Prevention of tumor progression in inflammation-related carcinogenesis by anti-inflammatory and anti-mutagenic effects brought about by ingesting fermented brown rice and rice bran with Aspergillus oryzae (FBRA). *Journal of Functional Foods*.

[44] Kapare H., Lohidasan S., Sinnathambi A., Mahadik K. (2019). Standardization, anti-carcinogenic potential and biosafety of Indian propolis. *Journal of Ayurveda and Integrative Medicine*.

[45] Izzo S., Naponelli V., Bettuzzi S. J. N. (2020). Flavonoids as epigenetic modulators for prostate cancer prevention. *Nutrients*.

[46] Shafique B., Mahmood S., Iqra I., Mustafa S. (2019). Anti-carcinogenic possessions of citrus peel extract and flavonoids. *Advanced Food and Nutritional Sciences*.

[47] Guven H., Arici A., Simsek O. (2019). Flavonoids in our foods: a short review. *Journal of Basic and Clinical Health Sciences*.

[48] Ren W., Qiao Z., Wang H., Zhu L., Zhang L. (2003). Flavonoids: promising anticancer agents. *Medicinal Research Reviews*.

[49] Farhadi F., Khameneh B., Iranshahi M., Iranshahy M. (2019). Antibacterial activity of flavonoids and their structure–activity relationship: an update review. *Phytotherapy Research*.

[50] Wang Z. F., Liu J., Yang Y. A., Zhu H. L. (2020). A review: the anti-inflammatory, anticancer and antibacterial properties of four kinds of licorice flavonoids isolated from licorice. *Current Medicinal Chemistry*.

[51] Comalada M., Ballester I., Bailón E. (2006). Inhibition of pro-inflammatory markers in primary bone marrow-derived mouse macrophages by naturally occurring flavonoids: analysis of the structure–activity relationship. *Biochemical Pharmacology*.

[52] Liao C. Y., Lee C. C., Tsai C. C. (2015). Novel investigations of flavonoids as chemopreventive agents for hepatocellular carcinoma. *BioMed Research International*.

[53] Calis Z., Mogulkoc R., Baltaci A. K. (2020). The roles of flavonols/flavonoids in neurodegeneration and neuroinflammation. *Mini Reviews in Medicinal Chemistry*.

[54] Devi S., Kumar V., Singh S. K., Dubey A. K., Kim J. J. (2021). Flavonoids: potential candidates for the treatment of neurodegenerative disorders. *Biomedicines*.

[55] Birla H., Minocha T., Kumar G., Misra A., Singh S. K. (2020). Role of oxidative stress and metal toxicity in the progression of Alzheimer’s disease. *Current Neuropharmacology*.

[56] Hwang S. L., Shih P. H., Yen G. C. (2012). Neuroprotective effects of citrus flavonoids. *Journal of Agricultural and Food Chemistry*.

[57] Wróbel-Biedrawa D., Grabowska K., Galanty A., Sobolewska D., Podolak I. (2022). A flavonoid on the brain: quercetin as a potential therapeutic agent in central nervous system disorders. *Life*.

[58] Silva dos Santos J., Gonçalves Cirino J. P., de Oliveira Carvalho P., Ortega M. M. (2021). The pharmacological action of kaempferol in central nervous system diseases: a review. *Frontiers in Pharmacology*.

[59] Rolt A., Cox L. S. (2020). Structural basis of the anti-ageing effects of polyphenolics: mitigation of oxidative stress. *Mitigation of Oxidative Stress*.

[60] Caro-Ordieres T., Marín-Royo G., Opazo-Ríos L. (2020). The coming age of flavonoids in the treatment of diabetic complications. *Journal of Clinical Medicine*.

[61] Rao R. V., Descamps O., John V., Bredesen D. E. (2012). Ayurvedic medicinal plants for Alzheimer's disease: a review. *Alzheimer's Research & Therapy*.

[62] Singh A. K., Gupta A., Mishra A. K., Gupta V., Bansal P., Kumar S. (2010). Medicinal plant for curing Alzheimer’s disease. *International Journal of Pharmaceutical & Biological Archive*.

[63] Obulesu M., Rao D. M. (2011). Effect of plant extracts on Alzheimer’s disease: an insight into therapeutic avenues. *Journal of neurosciences in rural practice*.

[64] Pradeep S., Jain A. S., Dharmashekara C. (2020). Alzheimer’s disease and herbal combination therapy: a comprehensive review. *Journal of Alzheimer's Disease Reports*.

[65] Onozuka H., Nakajima A., Matsuzaki K. (2008). Nobiletin, a citrus flavonoid, improves memory impairment and A*β* pathology in a transgenic mouse model of Alzheimer's disease. *Journal of Pharmacology and Experimental Therapeutics*.

[66] Wang J., Ho L., Zhao W. (2008). Grape-derived polyphenolics prevent A*β* oligomerization and attenuate cognitive deterioration in a mouse model of Alzheimer's disease. *Journal of Neuroscience*.

[67] Rezai-Zadeh K., Douglas Shytle R., Bai Y. (2009). Flavonoid-mediated presenilin-1 phosphorylation reduces Alzheimer's disease *β*-amyloid production. *Journal of Cellular and Molecular Medicine*.

[68] Wang Y.-J., Thomas P., Zhong J. H. (2009). Consumption of grape seed extract prevents amyloid-*β* deposition and attenuates inflammation in brain of an Alzheimer’s disease mouse. *Neurotoxicity Research*.

[69] Augustin S., Rimbach G., Augustin K., Schliebs R., Wolffram S., Cermak R. (2009). Effect of a short-and long-term treatment with Ginkgo biloba extract on amyloid precursor protein levels in a transgenic mouse model relevant to Alzheimer’s disease. *Archives of Biochemistry and Biophysics*.

[70] Mori T., Rezai-Zadeh K., Koyama N. (2012). Tannic acid is a natural *β*-secretase inhibitor that prevents cognitive impairment and mitigates Alzheimer-like pathology in transgenic mice. *Journal of Biological Chemistry*.

[71] Shimmyo Y., Kihara T., Akaike A., Niidome T., Sugimoto H. (2008). Epigallocatechin-3-gallate and curcumin suppress amyloid beta-induced beta-site APP cleaving enzyme-1 upregulation. *Neuroreport*.

[72] Vauzour D., Vafeiadou K., Rice-Evans C., Williams R. J., Spencer J. P. E. (2007). Activation of pro-survival Akt and ERK1/2 signalling pathways underlie the anti-apoptotic effects of flavanones in cortical neurons. *Journal of Neurochemistry*.

[73] Spencer J., Rice-Evans C., Williams R. (2003). Modulation of pro-survival Akt/PKB and ERK1/2 signalling cascades by quercetin and its in vivo metabolites. *Free Radical Biology and Medicine*.

[74] Nilsson P., Saido T. C. (2014). Dual roles for autophagy: degradation and secretion of Alzheimer's disease A*β* peptide. *BioEssays*.

[75] Kou X., Chen N. (2017). Resveratrol as a natural autophagy regulator for prevention and treatment of Alzheimer’s disease. *Nutrients*.

[76] Pierzynowska K., Podlacha M., Gaffke L. (2019). Autophagy-dependent mechanism of genistein-mediated elimination of behavioral and biochemical defects in the rat model of sporadic Alzheimer's disease. *Neuropharmacology*.

[77] Zaplatic E., Bule M., Shah S. Z. A., Uddin M. S., Niaz K. (2019). Molecular mechanisms underlying protective role of quercetin in attenuating Alzheimer's disease. *Life Sciences*.

[78] Zhu Y., Wang J. (2015). Wogonin increases *β*-amyloid clearance and inhibits tau phosphorylation via inhibition of mammalian target of rapamycin: potential drug to treat Alzheimer’s disease. *Neurological Sciences*.

[79] Song X., Liu B., Cui L. (2017). Silibinin ameliorates anxiety/depression-like behaviors in amyloid *β*-treated rats by upregulating BDNF/TrkB pathway and attenuating autophagy in hippocampus. *Physiology & Behavior*.

[80] Zhang X.-W., Chen J. Y., Ouyang D., Lu J. H. (2020). Quercetin in animal models of Alzheimer’s disease: a systematic review of preclinical studies. *International Journal of Molecular Sciences*.

[81] Cano A., Ettcheto M., Chang J. H. (2019). Dual-drug loaded nanoparticles of Epigallocatechin-3-gallate (EGCG)/ascorbic acid enhance therapeutic efficacy of EGCG in a APPswe/PS1dE9 Alzheimer's disease mice model. *Journal of Controlled Release*.

[82] Scholey A. B., French S. J., Morris P. J., Kennedy D. O., Milne A. L., Haskell C. F. (2010). Consumption of cocoa flavanols results in acute improvements in mood and cognitive performance during sustained mental effort. *Journal of Psychopharmacology*.

[83] Meyer H., Bolarinwa A., Wolfram G., Linseisen J. (2006). Bioavailability of apigenin from apiin-rich parsley in humans. *Annals of Nutrition and Metabolism*.

[84] Nielsen S. E., Young J. F., Daneshvar B. (1999). Effect of parsley (Petroselinum crispum) intake on urinary apigenin excretion, blood antioxidant enzymes and biomarkers for oxidative stress in human subjects. *The British Journal of Nutrition*.

[85] Zhang S., Tomata Y., Sugiyama K., Sugawara Y., Tsuji I. (2017). Citrus consumption and incident dementia in elderly Japanese: the Ohsaki cohort 2006 study. *British Journal of Nutrition*.

[86] Kean R. J., Lamport D. J., Dodd G. F. (2015). Chronic consumption of flavanone-rich orange juice is associated with cognitive benefits: an 8-wk, randomized, double-blind, placebo-controlled trial in healthy older adults. *The American journal of clinical nutrition*.

[87] Alex A. M., Arehally Marappa M., Joghee S., Chidambaram S. B. (2022). Therapeutic benefits of flavonoids against neuroinflammation: a systematic review. *Inflammopharmacology*.

[88] Singh S. S., Rai S. N., Birla H. (2020). Neuroprotective effect of chlorogenic acid on mitochondrial dysfunction-mediated apoptotic death of DA neurons in a Parkinsonian mouse model. *Oxidative Medicine and Cellular Longevity*.

[89] Birla H., Keswani C., Singh S. S. (2021). Unraveling the neuroprotective effect of Tinospora cordifolia in a Parkinsonian mouse model through the proteomics approach. *ACS Chemical Neuroscience*.

[90] Gu X.-H., Xu L. J., Liu Z. Q. (2016). The flavonoid baicalein rescues synaptic plasticity and memory deficits in a mouse model of Alzheimer’s disease. *Behavioural Brain Research*.

[91] Szwajgier D. (2015). Anticholinesterase activity of selected phenolic acids and flavonoids-interaction testing in model solutions. *Annals of Agricultural and Environmental Medicine*.

[92] Strittmatter W. J., Saunders A. M., Schmechel D. (1993). Apolipoprotein E: high-avidity binding to beta-amyloid and increased frequency of type 4 allele in late-onset familial Alzheimer disease. *Proceedings of the National Academy of Sciences*.

[93] Mahley R. W., Weisgraber K. H., Huang Y. (2006). Apolipoprotein E4: a causative factor and therapeutic target in neuropathology, including Alzheimer’s disease. *Proceedings of the National Academy of Sciences*.

[94] Nistor M., Don M., Parekh M. (2007). Alpha- and beta-secretase activity as a function of age and beta-amyloid in Down syndrome and normal brain. *Neurobiology of Aging*.

[95] Hardy J., Allsop D. (1991). Amyloid deposition as the central event in the aetiology of Alzheimer's disease. *Trends in Pharmacological Sciences*.

[96] Bachman D., Wolf P. A., Linn R. (1992). Prevalence of dementia and probable senile dementia of the Alzheimer type in the Framingham study. *Neurology*.

[97] Uriarte-Pueyo I., Calvo M. I. (2011). Flavonoids as acetylcholinesterase inhibitors. *Current Medicinal Chemistry*.

[98] Orhan I., Kartal M., Tosun F., Şener B. (2007). Screening of various phenolic acids and flavonoid derivatives for their anticholinesterase potential. *Zeitschrift für Naturforschung C*.

[99] Polvikoski T., Sulkava R., Haltia M. (1995). Apolipoprotein E, dementia, and cortical deposition of *β*-amyloid protein. *New England Journal of Medicine*.

[100] Lacor P. N., Buniel M. C., Furlow P. W. (2007). A*β* oligomer-induced aberrations in synapse composition, shape, and density provide a molecular basis for loss of connectivity in Alzheimer's disease. *Journal of Neuroscience*.

[101] Nikolaev A., McLaughlin T., O’Leary D. D. M., Tessier-Lavigne M. (2009). APP binds DR6 to trigger axon pruning and neuron death via distinct caspases. *Nature*.

[102] Turner P. R., O’Connor K., Tate W. P., Abraham W. C. (2003). Roles of amyloid precursor protein and its fragments in regulating neural activity, plasticity and memory. *Progress in Neurobiology*.

[103] Goedert M., Spillantini M., Crowther R. (1991). Tau proteins and neurofibrillary degeneration. *Brain Pathology*.

[104] Mudher A., Lovestone S. (2002). Alzheimer's disease - do tauists and baptists finally shake hands?. *Trends in Neurosciences*.

[105] Iqbal K., del C Alonso A., Chen S. (2005). Tau pathology in Alzheimer disease and other tauopathies. *Biochimica et Biophysica Acta (BBA)-Molecular Basis of Disease*.

[106] Krikorian R., Nash T. A., Shidler M. D., Shukitt-Hale B., Joseph J. A. (2010). Concord grape juice supplementation improves memory function in older adults with mild cognitive impairment. *British Journal of Nutrition*.

[107] Tikhonova M. A., Tikhonova N. G., Tenditnik M. V. (2020). Effects of grape polyphenols on the life span and neuroinflammatory alterations related to neurodegenerative parkinson disease-like disturbances in mice. *Molecules*.

[108] Kent K., Charlton K., Roodenrys S. (2017). Consumption of anthocyanin-rich cherry juice for 12 weeks improves memory and cognition in older adults with mild-to-moderate dementia. *European Journal of Nutrition*.

[109] Hartman R. E., Shah A., Fagan A. M. (2006). Pomegranate juice decreases amyloid load and improves behavior in a mouse model of Alzheimer's disease. *Neurobiology of Disease*.

[110] Kametani F., Hasegawa M. (2018). Reconsideration of amyloid hypothesis and tau hypothesis in Alzheimer's disease. *Frontiers in Neuroscience*.

[111] Shukitt-Hale B., Cheng V., Joseph J. A. (2009). Effects of blackberries on motor and cognitive function in aged rats. *Nutritional Neuroscience*.

[112] Shih P. H., Chan Y. C., Liao J. W., Wang M. F., Yen G. C. (2010). Antioxidant and cognitive promotion effects of anthocyanin-rich mulberry (Morus atropurpurea L.) on senescence-accelerated mice and prevention of Alzheimer's disease. *The Journal of Nutritional Biochemistry*.

[113] El-Shiekh R. A., Ashour R. M., Abd El-Haleim E. A., Ahmed K. A., Abdel-Sattar E. (2020). Hibiscus sabdariffa L.: A potent natural neuroprotective agent for the prevention of streptozotocin-induced Alzheimer 's disease in mice. *Biomedicine & Pharmacotherapy*.

[114] Lee A. Y., Choi J. M., Lee Y. A., Shin S. H., Cho E. J. (2020). Beneficial effect of black rice (Oryza sativa L. var. japonica) extract on amyloid *β*-induced cognitive dysfunction in a mouse model. *Experimental and Therapeutic Medicine*.

[115] Tikhonova M. A., Shoeva O. Y., Tenditnik M. V. (2020). Evaluating the effects of grain of isogenic wheat lines differing in the content of anthocyanins in mouse models of neurodegenerative disorders. *Nutrients*.

[116] Desikan R. S., Cabral H. J., Hess C. P. (2009). Automated MRI measures identify individuals with mild cognitive impairment and Alzheimer's disease. *Brain*.

[117] Tiraboschi P., Hansen L. A., Thal L. J., Corey-Bloom J. (2004). The importance of neuritic plaques and tangles to the development and evolution of AD. *Neurology*.

[118] Mendez M. F. (2006). The accurate diagnosis of early-onset dementia. *The International Journal of Psychiatry in Medicine*.

[119] Waldemar G., Dubois B., Emre M. (2007). Recommendations for the diagnosis and management of Alzheimer's disease and other disorders associated with dementia: EFNS guideline. *European Journal of Neurology*.

[120] National Collaborating Centre for Mental Health (2011). *Dementia: supporting people with dementia and their carers in health and social care*.

[121] Schroeter M. L., Stein T., Maslowski N., Neumann J. (2009). Neural correlates of Alzheimer's disease and mild cognitive impairment: a systematic and quantitative meta-analysis involving 1351 patients. *NeuroImage*.

[122] Nijveldt R. J., van Nood E., van Hoorn D. E. C., Boelens P. G., van Norren K., van Leeuwen P. A. M. (2001). Flavonoids: a review of probable mechanisms of action and potential applications. *The American Journal of Clinical Nutrition*.

[123] Akter R., Chowdhury M. A., Rahman M. H. (2021). Flavonoids and polyphenolic compounds as potential talented agents for the treatment of Alzheimer’s disease and their antioxidant activities. *Current Pharmaceutical Design*.

[124] Singh S. K., Gaur R., Kumar A., Fatima R., Mishra L., Srikrishna S. (2014). The flavonoid derivative 2-(4′ benzyloxyphenyl)-3-hydroxy-chromen-4-one protects against A*β*42-induced neurodegeneration in transgenic drosophila: insights from in silico and in vivo studies. *Neurotoxicity Research*.

[125] Singh S. S., Rai S. N., Birla H., Zahra W., Rathore A. S., Singh S. P. (2020). NF-*κ*B-mediated neuroinflammation in Parkinson’s disease and potential therapeutic effect of polyphenols. *Neurotoxicity Research*.

[126] Zahra W., Rai S. N., Birla H. (2020). Neuroprotection of rotenone-induced Parkinsonism by ursolic acid in PD mouse model. *CNS & Neurological Disorders-Drug Targets (Formerly Current Drug Targets-CNS & Neurological Disorders)*.

[127] Birla H., Rai S. N., Singh S. S. (2019). Tinospora cordifolia suppresses neuroinflammation in parkinsonian mouse model. *Neuromolecular Medicine*.

[128] Ferrali M., Signorini C., Caciotti B. (1997). Protection against oxidative damage of erythrocyte membrane by the flavonoid quercetin and its relation to iron chelating activity. *FEBS Letters*.

[129] Kumar S., Pandey A. K. (2013). Chemistry and biological activities of flavonoids: an overview. *The Scientific World Journal*.

[130] Lotito S. B., Frei B. (2006). Consumption of flavonoid-rich foods and increased plasma antioxidant capacity in humans: cause, consequence, or epiphenomenon?. *Free Radical Biology and Medicine*.

[131] Tang Z., Zhang Q. (2022). The potential toxic side effects of flavonoids. *Biocell*.

[132] Patisaul H. B. (2017). Endocrine disruption by dietary phyto-oestrogens: impact on dimorphic sexual systems and behaviours. *Proceedings of the Nutrition Society*.

[133] Prasain J. K., Wang C. C., Barnes S. (2004). Mass spectrometric methods for the determination of flavonoids in biological samples. *Free Radical Biology & Medicine*.

[134] Dunnick J. K., Halley J. R. (1992). Toxicity and carcinogenicity studies of quercetin, a natural component of foods. *Toxicological Sciences*.

[135] Kandar C. C. (2022). Herbal flavonoids in healthcare. *Herbal Biomolecules in Healthcare Applications*.

[136] Kiskova T., Kubatka P., Büsselberg D., Kassayova M. (2020). The plant-derived compound resveratrol in brain cancer: a review. *Biomolecules*.

[137] Baell J., Walters M. A. (2014). Chemistry: chemical con artists foil drug discovery. *Nature*.

[138] Ingolfsson H. I., Thakur P., Herold K. F. (2014). Phytochemicals perturb membranes and promiscuously alter protein function. *ACS Chemical Biology*.

[139] Doll R., Peto R. (1981). The causes of cancer: quantitative estimates of avoidable risks of cancer in the United States today. *JNCI: Journal of the National Cancer Institute*.

[140] Macgregor J. T., Jurd L. (1978). Mutagenicity of plant flavonoids: structural requirements for mutagenic activity in Salmonella typhimurium. *Mutation Research/Environmental Mutagenesis and Related Subjects*.

[141] Hodnick W. F., Kung F. S., Roettger W. J., Bohmont C. W., Pardini R. S. (1986). Inhibition of mitochondrial respiration and production of toxic oxygen radicals by flavonoids: a structure-activity study. *Biochemical Pharmacology*.

